# Synthesis of size-controlled monodisperse Pd nanoparticles via a non-aqueous seed-mediated growth

**DOI:** 10.1186/1556-276X-7-312

**Published:** 2012-06-19

**Authors:** Lei Zhang, Lin Wang, Zhiyuan Jiang, Zhaoxiong Xie

**Affiliations:** 1 State Key Laboratory of Physical Chemistry of Solid Surfaces, College of Chemistry and Chemical Engineering, Xiamen University, Xiamen, 361005, China; 2 Department of Chemistry, College of Chemistry and Chemical Engineering, Xiamen University, Xiamen, 361005, China

**Keywords:** Nanocrystalline materials, Crystal growth, Pd, Non-aqueous seed-mediated growth, Self-assembly

## Abstract

We demonstrated that stepwise seed-mediated growth could be extended in non-aqueous solution (solvothermal synthesis) and improved as an effective method for controlling the uniform size of palladium nanoparticles (Pd NPs) in a wide range. The monodisperse Pd NPs with the size of about 5 nm were synthesized by simply reducing Pd(acac)_2_ with formaldehyde in different organic amine solvents. By an improved stepwise seed-mediated synthesis, the size of the monodisperse Pd NPs can be precisely controlled from approximately 5 to 10 nm. The as-prepared Pd NPs could self assemble to well-shaped superlattice crystal without size selection process.

## Background

As an important catalyst and a promising alternative of Pt-based catalysts, Pd has received continual interest in the past decades [1-4]. For example, Pd nanoparticles (Pd NPs) can be used as efficient catalysts for a large number of carbon-carbon coupling reactions [5,6] as well as hydrogenation/dehydrogenation reactions [7,8]. Pd NPs have also been demonstrated to exhibit efficient electrocatalytic activities in fuel cell reactions [9,10]. As one of the solid heterogeneous catalysts, the performance of Pd strongly depends on the size and/or shape, especially in nanometer scale [11,12]. Therefore, synthesis of monodisperse Pd NPs with uniform shape has been an important subject of chemical research.

Many methods, especially solution-based synthesis with various protecting agents, have been developed to prepare monodisperse Pd NPs [13-15]. However, most of the reported capping agents, which were used to stabilize Pd NPs and prevent their aggregation, have drawbacks under harsh catalytic reaction conditions because a ‘clean’ surface of the nanoparticles was difficult to get due to the presence of bulky or strongly binding surfactants around these nanoparticles [16]. Recently, several groups have succeeded in synthesizing monodisperse Pd NPs with a size smaller than 5 nm by applying oleylamine as the capping agent [17,18]. However, it remains a challenge to control the size of the Pd NPs in a wider range while keeping their uniformity.

Seed-mediated growth was widely adopted in synthesis of nanoparticles with different size, but most of them were applied in aqueous solution [19-22]. There were few research reports concerning the seed-mediated growth in non-aqueous solution. In this communication, the stepwise seed-mediated growth was extended in non-aqueous solution (solvothermal synthesis) and improved as an effective method for controlling the uniform size of Pd NPs in a wide range. The seeds were prepared by simply reducing palladium(II) acetylacetonate (Pd(acac)_2_) with formaldehyde in different organic amine solvents. The size of the monodisperse Pd NPs can be precisely controlled from about 5 to 10 nm. In addition, we concluded that amine groups played critical role in stabilizing the Pd NPs.

## Methods

### Materials

The synthesis was carried out using commercially available reagents. Palladium(II) acetylacetonate, octadecylamine (98%) and n-octylamine (99%) were purchased from Alfa Aesar (Beijing, China), while oleylamine (80% to 90%) was from Acros Organics (Shanghai, China). Butylamine and formaldehyde were purchased from Sinopharm Chemical Reagent Co. Ltd. (Shanghai, China). All chemicals were used as received without further purification.

### Synthesis of 5-nm monodisperse Pd NPs

Typically, 5.0 mg Pd(acac)_2_ was dissolved in the mixture of 10 mL of organic amine (such as oleylamine, octadecylamine, n-octylamine and butylamine) and 0.2 mL formaldehyde to form growth solution. The growth solution was then transferred to a 25-mL Teflon-lined autoclave and maintained at 200°C for 3 h. The product (about 5 nm Pd NPs) was separated by centrifugation (12,000 rpm for 10 min). The product was then dispersed in hexane.

### Stepwise seed-mediated solvothermal growth

As for the preparation of Pd NPs of about 8 nm, the as-prepared 5-nm Pd NP solution without separation was employed as the seed, and a seed-mediated growth was carried out by mixing 2-mL seed solution and the growth solution, followed by solvothermal treatment at 200°C for 3 h. Similarly, about 10-nm Pd NPs were synthesized using unseparated 8-nm Pd NPs as growth seeds by keeping the same growth condition.

### Structural characterizations

The morphology and structure of the products were characterized by scanning electron microscope (SEM, Hitachi S-4800, Japan), transmission electron microscope (TEM, JEM-2100, Japan) and X-ray powder diffraction (XRD, Panalytical X-pert diffractometer with Cu-Kα radiation; PANalytical B.V., Netherlands). The samples for IR spectra measurements (Nicolet 330) were obtained by depositing the ethanol suspensions of Pd NPs on KBr substrate followed by solvent evaporation.

## Results and discussion

**Figure 1:**
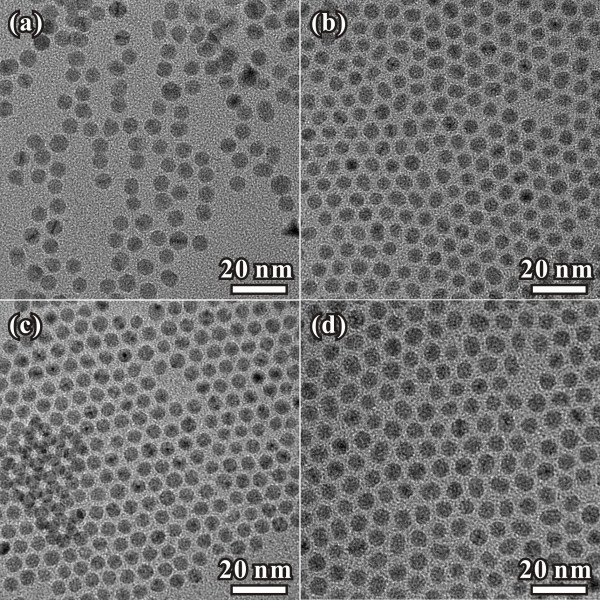
**TEM images of monodisperse Pd nanoparticles prepared in different organic amines.** (**a**) Oleylamine, (**b**) octadecylamine, (**c**) n-octylamine and (**d**) butylamine.

Figure 1a-d shows the representative TEM images of Pd NPs prepared using oleylamine, octadecylamine, n-octylamine and butylamine, respectively. It can be seen that all of the samples consist of Pd NPs with a uniform size. These nanoparticles tended to arrange in a two-dimensionally hexagonal close-packed array due to their high uniformity. The average sizes of the as-prepared particles are 5.4, 5.0, 4.7 and 5.8 nm, respectively. By comparing the Pd NPs prepared in oleylamine or octadecylamine, no obvious difference can be observed, indicating that the formation of the Pd NPs is not affected by the double bond in oleylamine. With the chain length of the organic amines changing from eighteen to four, the size of Pd NPs increase from about 5 to 5.8 nm, which may be caused by the weaker protection of the organic amines with shorter chain length. The result indicated that the organic amines performed as the capping-agent to protect the Pd NPs from aggregation. It should be pointed that a proper reduction rate would play a key role for the preparation of NPs with a narrow size distribution. A strong reducing agent (e.g., NaBH_4_, LiBH_4_) makes the nucleating process very fast, so it is difficult to obtain NPs with a uniform size [23]. Many reports showed that a weaker reductant, such as amine-borane complexes, was suitable for the syntheses of monodisperse metallic NPs [17,18,23]. In the present case, formaldehyde was chosen as the reducing agent, and the products showed that it also performed an effective reducing rate for the preparation of Pd NPs with a narrow size distribution.

**Figure 2:**
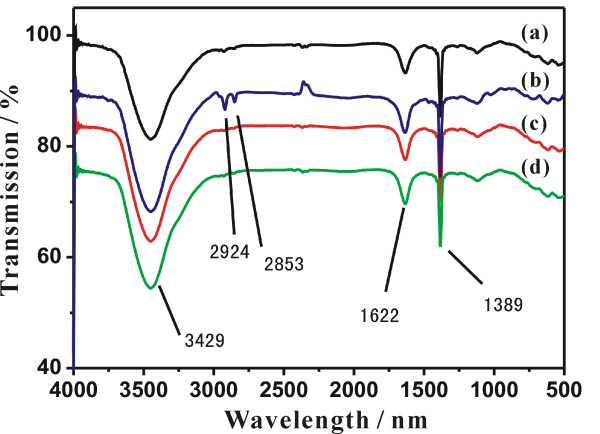
**FT-IR spectra of the samples.** Samples were prepared in (**a**) oleylamine, (**b**) octadecylamine, (**c**) n-octylamine and (**d**) butylamine.

To reveal the growth mechanism of the as-prepared Pd NPs, the Fourier transform infrared (FT-IR) was employed to investigate the stabilizing ligands existed in the surface of the Pd NPs for stabilizing ligands or capping agents usually play a key role in the formation of monodisperse nanocrystals. In the FT-IR spectra of the as-prepared Pd NPs (Figure 2), the peak located at about 1389 cm^−1^ is assigned to C-N stretching, indicating the presence of organic amine. The peaks located at about 2,924 and 2,853 cm^−1^ are assigned to C-H symmetric and asymmetric stretching of methylene. The peak located at about 3,429 cm^−1^ is assigned to N-H stretching, and 1,622 cm^−1^ to N-H rocking vibrations of the organic amines [24]. It should be pointed that an N-H stretching of free amines should locate at about 3,400 cm^−1^. The peak, which shifted to 3,429 cm^−1^, revealed the covalent bonding of the N-H to the Pd surface. These FT-IR spectra provided supportive evidence that organic amine exactly existed on the surface of the as-prepared monodisperse Pd NPs [24]. Therefore, we speculate that organic amine performs as a solvent and the stabilizer for formation of monodisperse Pd NPs. Formaldehyde was used to control the reacting rate which made the system tend to nucleate gently.

**Figure 3:**
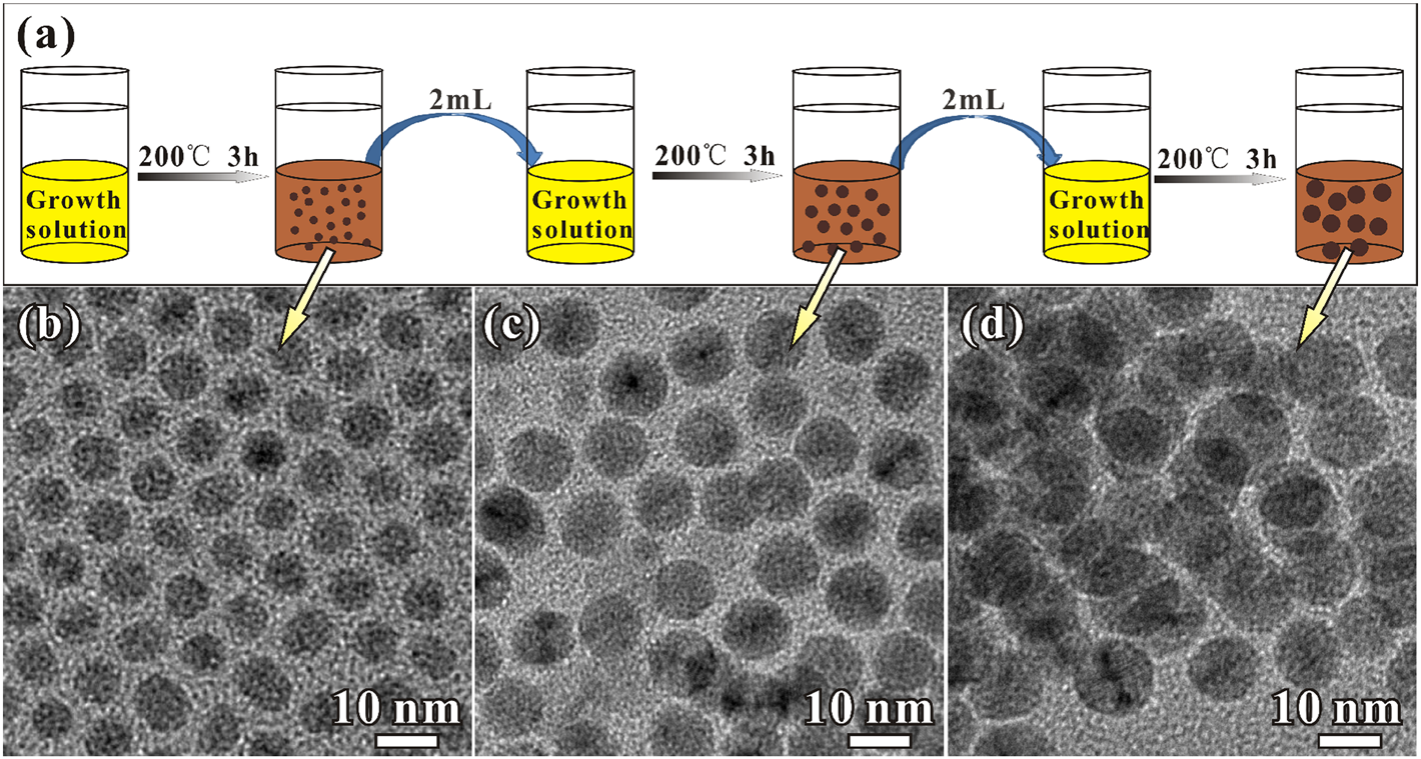
**Synthetic procedure and TEM images and size distribution of Pd NPs.** (**a**) Schematic illustration of the synthetic procedure used for the preparation of monodisperse Pd NPs with different sizes by stepwise seed-mediated solvothermal growth. TEM images of Pd NPs prepared after the first (**b**), second (**c**) and third growths (**d**).

The size of the Pd NPs could be easily controlled by the stepwise seed-mediated growth in non-aqueous solution. Instead of H_2_O applied in the aqueous seed-mediated growth for the syntheses of Au and Pd NPs. The mixture of formaldehyde and organic amine was used as the solvent in the present study. The Pd NPs prepared in the organic amine solvents could be conveniently employed as the seed for further growth due to their uniform size and good dispersion. Figure 3a illustrated the synthetic procedure for the stepwise seed-mediated solvothermal growth. The monodisperse Pd NPs prepared in n-octylamine were employed as the seeds for the stepwise growth in the mixture of Pd(acac)_2_ (5.0 mg), n-octylamine (10 mL) and formaldehyde (0.2 mL). Thus, the Pd NPs could grow bigger and bigger after solvothermal process again and again. Figure 3b-d shows the TEM images and size distribution of Pd NPs prepared via second and third growths. The average sizes of the Pd NPs have been finely tuned to 7.8 and 9.5 nm, while the uniformity was well kept. It should be pointed out that the growth parameters, such as the concentration of Pd(acac)_2_, the temperature and the reaction time, should be well controlled in these seed-mediated growth to avoid new nuclear phenomenon, especially in further growth circles. Otherwise, the size of products will be in a larger range.

**Figure 4:**
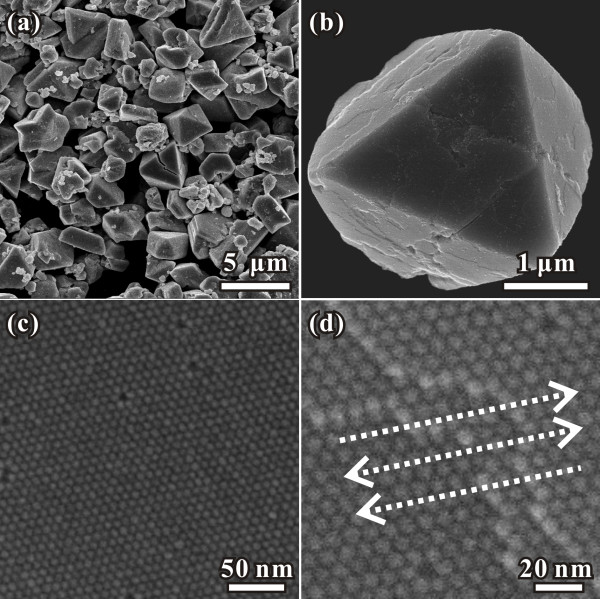
**Typical SEM image of the 3-D superlattice of Pd NPs.** (**a**) SEM image of superlattice crystals formed by as-prepared Pd NPs with the size of about 8 nm, (**b**) single superlattice crystal with octahedron shape, (**c**) exposed surfaces arranged by close-packed nanocrystals, (**d**) magnified step surface.

The uniformity of as-prepared Pd NPs can also be confirmed by their self-assemble three-dimensional superlattices. As it is well known, a narrow particle size distribution is required to obtain long-range superlattice ordering. Just by diffusing 0.2-mL ethanol into the reacted mixture, micrometer-sized 3-D superlattice crystals could be easily prepared in the present case without further size selection process. Figure 4a shows the SEM image of superlattice crystals formed by as-prepared Pd NPs with an average size of 7.8 nm, indicating the formation of well-shaped crystals with a size of several micrometers, and most of them were octahedron or distorted octahedron. Figure 4b is a single superlattice crystal with octahedron shape, all of the exposed surfaces (Figure 4c) were arranged by close-packed nanocrystals similar with the atom arrangement in the {111} surfaces of face-centered cubic (fcc) metal crystals. A closer look of some step surface (Figure 4d) revealed that crystals were self assembled by close-packed monodisperse Pd nanocrystals in the sequence of ABCABC…, and thus formed an fcc structure. In the formation of superlattice crystals, the individual nanoparticles can be considered as ‘artificial atoms’ as they can exhibit atomic-like behaviors [25,26]. Just as commonly observed in the fcc metal nanocrystals, {111} surface was often exposed to minimize the total surface energy. In the present case, the {111} surfaces were also exposed in this concept; thus, the predominate morphologies were octahedron or distorted octahedron.

**Figure 5:**
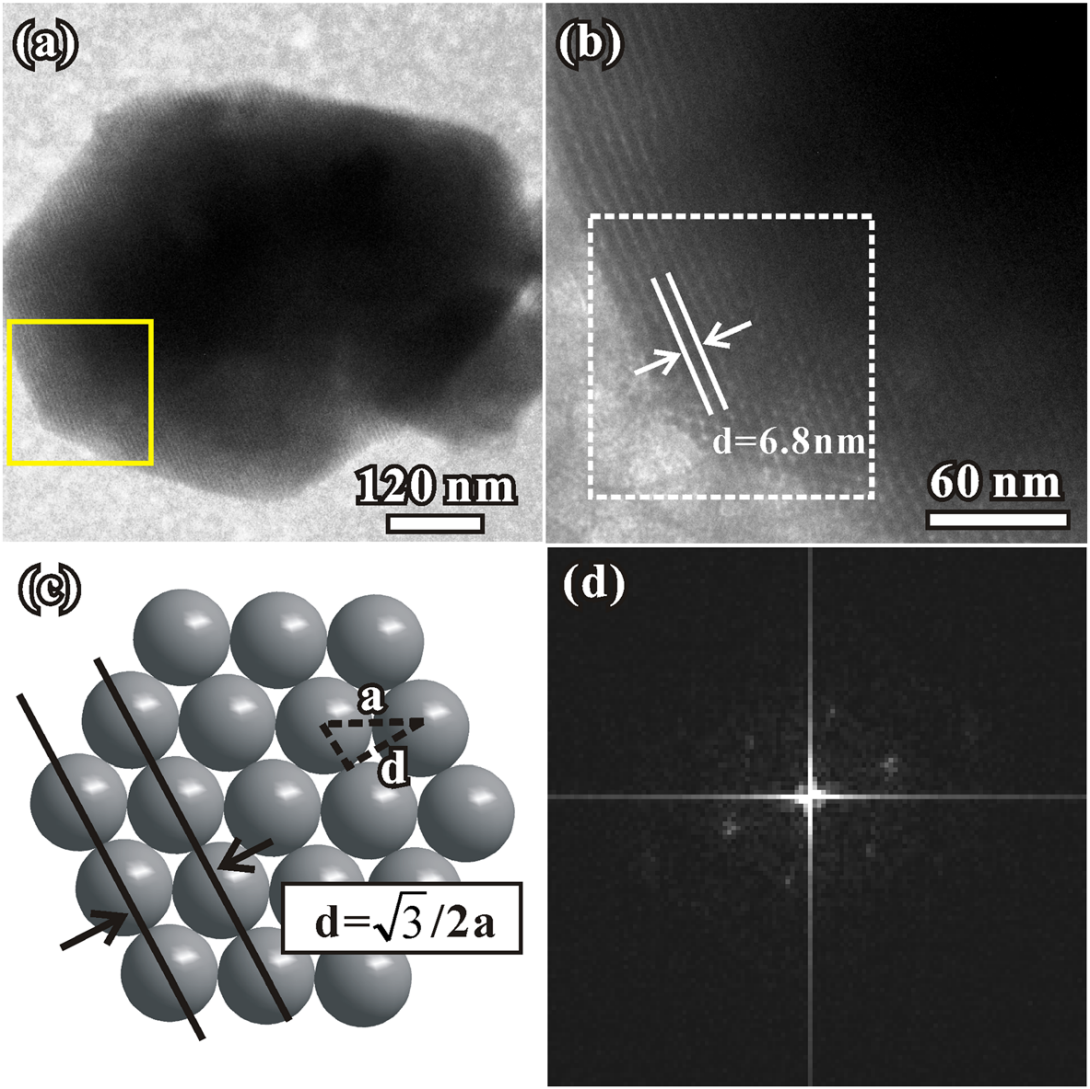
**TEM images, atomic model and FFT pattern.** (**a**) Typical TEM images of the 3-D superlattice of Pd NPs with the size of about 8 nm. (**b**) TEM image of the region indicated by the box in (**a**). (**c**) Atomic model with the close-packed structure. (**d**) FFT pattern of the region indicated by the box in (**b**).

The fcc structure was further proved in the TEM analysis. Figure 5a shows the TEM image of one typical superlattice crystals. Figure 5b is an enlarged TEM image from the region indicated by the box in Figure 5a. The distance between two rows of NPs is 6.8 nm, which is 0.87 times to the size of the NPs. Figure 5c shows the atomic model with close-packed structure. The lattice distance is $\sqrt{3}/2$ to the diameter of the atoms. The data we measured in the TEM image matched the atomic model well, which further proved the close-packed structure of the superlattice. The corresponding FFT pattern (Figure 5d) is similar with selected area electron diffraction pattern of fcc structure along [111] zone axis, indicating that the 3-D superlattice crystals are almost perfectly aligned according to the fcc close-packed structure. These results are in good agreement with the information gotten from the SEM images. Understanding of sedimentation and aggregation behavior of NPs would be significant to predict their environmental transport and fate [27]. The self-assemble phenomenon observed in the present case may also helpful for the study of further application.

## Conclusions

In summary, monodisperse Pd NPs were prepared via a facile solvothermal synthesis using organic amine as solution and stabilizing agent. The size of the Pd NPs could be controlled from about 5 to 10 nm by an improved stepwise seed-mediated method. The as-prepared Pd nanoparticles could self assemble to well-shaped superlattice crystal without size selection process. Organic amine was proved to play a critical role in the formation of monodisperse Pd NPs.

## Abbreviations

FT-IR: Fourier transform infrared; Pd(acac)_2_ :palladium(II) acetylacetonate; Pd NPs: Pd nanoparticles; SEM: scanning electron microscopy; TEM: transmission electron microscope.

## Competing interests

The authors declare that they have no competing interests.

## Authors’ contributions

LZ and ZJ conceived and designed the experiments, analyzed the data and co-wrote the paper. LW performed some of the electron microscopy experiments. ZX gave the advice and guided the research. All authors discussed the results, read and approved the final manuscript.
